# Acknowledgment to Reviewers of *Bioengineering* in 2020

**DOI:** 10.3390/bioengineering8020015

**Published:** 2021-01-20

**Authors:** Bioengineering Editorial Office

**Affiliations:** MDPI AG, St. Alban-Anlage 66, 4052 Basel, Switzerland

Peer review is the driving force of journal development, and reviewers are gatekeepers who ensure that *Bioengineering* maintains its standards for the high quality of its published papers. Thanks to the cooperation of our reviewers, in 2020, the median time to first decision was 39 days and the median time to publication was 17 days. The editors would like to express their sincere gratitude to the following reviewers for their precious time and dedication, regardless of whether the papers were finally published:
Abdullah, AdilMartinelli, AndreaAhn, Chi BumMartinez-Garcia, Mari CarmenAiello, OrazioMartins, Ramiro J.E.Akkineni, Ashwini RahulMartucci, GennaroAlbaugh, VanceMaspero, CinziaAli, Moustafa R.K.Massai, DianaAllardyce, BenMasters, KristynAlquié, GeorgesMata, ÁlvaroAltmann, FriedrichMatharu, Rupy KaurAmini, RouzbehMauk, Michael G.Angelucci, EmanueleMaurizi, LionelAranda, AgustínMcclure, Michael J.Arul, MichaelMcKay, TinaAuxillos, JamieMehrban, NaziaAvazmohammadi, RezaMerrin, JackAw, RochelleMichailos, StavrosAydin, RolandMiftahof, RoustemBach, Thomas J.Mikulski, DawidBago, Juli R.Miniaci, Maria ConcettaBaig, Khurram ShahzadMinutti, CarlosBalusamy, BrabuMironov, VladimirBar-Nur, OriMishra, AlaknandaBarry, Michael A.Mitsunaga, ShuichiBasaglia, MarinaMolina-Espeja, PatriciaBashashati, MohammadMomeny, MajidBasnett, PoojaMondal, SudipBauser-Heaton, HollyMoradshahi, MehradBazrafshan, ZahraMoreno, AntonioBegines, BelenMoreno, Antonio D.Bellato, AlessioMorrison, Wayne A.Benjdia, AlhosnaMostaço-Guidolin, LeilaBerjano-Zanón, EnriqueMozejko-Ciesielska, JustynaBertasi, GiampietroMügge, CarolinBhatia, Shashi KantMulvihill, JohnBirzer, Cristian H.Murab, SumitBlunt, WarrenNakashimada, YutakaBoca, SandaNalakath, HarisBrahmbhatt, BhaumikNamer, BarbaraBrey, Eric M.Naoto, ShimizuBrzeszczynska, JoannaNappi, FrancescoBuehler, KatjaNarayanan, BarathBueno-Silva, BrunoNascimento, Luís Adriano Santos DoBurpo, FredNaser, M.Z.Calitz, CarlemiNashimoto, YujiCampo, GiuseppeNath, AudreyCao, ZehongNazaruk, EwaCarere, CarloNicholson, John W.Carniel, EmanueleNienow, Alvin WCarrère, HélèneNoiri, YuichiroCarter, Dee A.Novitskiy, Sergey V.Caseiro, Ana RitaNowak, Agnieszka J.Cassani, RaymundoObrist, DominikCasteleijn, MarcoOchia, RuthCatalan, MarceloOh, Seh-HoonCengiz, Ibrahim FatihOlson, MichaelCereda, MatteoOlvera, DinorathCervigón, RaquelOrtega, IlidaChablat, Damien CharlesOtani, TomohiroChagnon, GregoryOury, CécileChakraborty, AtanuOuyang, LiliangChamuleau, Robert A.F.M.Pabelick, Christina M.Chan, AlexPacelli, SettimioChaudhry, RasulPacheco, AdrianaCheng, ChengPadberg, Frank TChevali, VenkataPaez, PaulinaChiulan, IoanaPal, AjayChiumenti, AlessandroPal, DhananjayChoo, Hyo-jungPalchesko, RachelleChoudhury, DeepakPałka, KrzysztofChow, Lesley W.Pandya, AbhilashChu, Chen-YeonPapantoniou, IoannisCoelho, LuisPappa, Anna MariaComeau, PatriciaParashar, DeepakConnon, CheParekh, SapunCorbella, StefanoParente, MarcoCosentino, CarloPark, Chan HeeCristache, Corina MarilenaPark, InsuCrook, NathanPatnaik, Sourav S.Cruciani, SaraPaulauskaite-Taraseviciene, AgneCsapó, EditPaulus, Yannis M.Cui, WenguoPecci, RaffaellaCunnane, EoghanPeibiao, ZhangDallon, JohnPekkanen-Mattila, MariDas, SudipPelzl, LisannDash, BirajaPennisi, Cristian PabloDavid, Madalina ElenaPérez, RocíoDavoodi, PooyaPerlingeiro, Rita C.R.De Greef, Jessica C.Persikov, AntonDelgado-Gonzalo, RicardPetrov, TatjanaDell’Amore, AndreaPhan, Thi Tuong VyDi Girolamo, StefanoPiegat, AgnieszkaDi Micco, LuigiPierce, David M.Diamantis, Vasileios I.Pins, George D.Díaz, EsperanzaPiperigkou, ZoiDikicioglu, DuyguPipino, CaterinaDing, ZiyunPiras, CarmenDmitrieva, Natalia I.Piscioneri, AntonellaDoblaré, ManuelPolese, LinoDobrev, IvoPontremoli, CarlottaDocea, Anca OanaPopovic, ZoyaDodi, GianinaPowell, HeatherDonadu, Matthew GavinoPozzobon, MichelaDumont, JosephPrehn-Kristensen, AlexanderDunlop, DouglasPrentice, MichaelDutton, James R.Pribilova, AnnaDybowska-Sarapuk, LucjaRafael, Nicolosi LibanoriEchauri, Sergio GarciaRaghavan, ShreyaEhman, Nanci VanesaRaines, Ronald T.Engelhard, Herbert H.Rajagopal, RajinikanthEnrione, JavierRamanathan, GiriprasathEspa, StefaniaRamstrand, NerrolynEspinosa, EduardoRanjan, Amalendu P.Estacion, MarkRanjan, BibhutiEsteve Mas, Maria JoséRao, SmithaFederspiel, William J.Rashdan, NabilFedorov, AndreyRedon, PauFehr, AndréRen, XiFeng, PeiRenukuntla, JwalaFernandes, TiagoRestaino, Odile FrancescaFernandes-Platzgummer, AnaRiedel, Sebastian L.Ferreira, Antonio Joaquim MendesRinaldi, AntonioFishbein, IliaRios Solis, LeonardoForlani, FabioRoberts-Galbraith, RachelFossum, MagdalenaRodrigo, MiguelFrancis-Boyle, OliviaRodrigues, CarlosFrattaruolo, BrLucaRodrigues, JoanaFridman, Gene YevgenyRodrigues, RaquelFue, KadegheRodriguez, Ruben J. SanchezFukumoto, MasahiroRodríguez, Ricardo ReyesGajavelli, ShyamRoehri, NicolasGarcia, Jose L.Romanach, RodolfoGarcia-Alias, GuillermoRomanos, GeorgiosGarcia-Ruiz, EvaRomero, AlbertoGarg, KoyalRomero-Uribe, GabrielaGeissler, SvenRomo, LuciaGhosh, ArijitRösel, DanielGiuseppe, BaselliRossi, SimoneGlattauer, VeronicaRowland, TeishaGomes, DanielRuggiero Braga, RobertoGoršek, AndrejaRybicki, FrankGouveia, RicardoSahoo, Jugal KishoreGraczyk-Jarzynka, AgnieszkaSakaguchi, DonaldGrasman, JonathanSalehi, SarahGray, MichaelSam, Christian AhobaGrazioso, StanislaoSambri, AndreaGreene, BarrySanabria, JanethGreising, Sarah M.Sanz, Jesus MGrosser, AnnaSapudom, JiranuwatGrunsven, Leo VanSasmal, AniruddhaGrząbka-Zasadzińska, AleksandraSass, F AndreaGuo, JunlingSaveleva, MariiaGupta, AkashScaramuzza, MatteoHaïkel, YoussefScheller, SilvanHan, Dong-WookSchwaminger, SebastianHan, PingpingSchweizer, MichaelHandral, HarishScotto Di Perta, EsterHatoum, HodaSedlacek, PetrHaubruck, PatrickSELLES, BenjaminHe, XiaomingSen, ChandaniHermenean, AncaSeo, Ji-WonHirsch, OliverSerpooshan, VahidHnatiuc, MihaelaSerrancolí, GilHoang, Ngoc HaSevilimedu Veeravalli, SathyanarayananHolzapfel, Gerhard A.Shankles, PeterHomem, NatáliaShao, ShuaiHong, YiSharbeen, GeorgeHsu, Fu-YinSharfstein, SusanHuang, PengSharma, AshutoshHuang, WeichenShavandi, AminBennett, Hunter J.Shea, Richard O.Leo, Hwa LiangSheppard, PhilHwang, ChangmoShi, JunweiIliescu, CiprianShishatskaya, Ekaterina IgorevnaInaba, MasafumiShopova, DobromiraIngrassia, TommasoShrestha, NamitaIyer, Arun K.Sikavitsas, VassiliosJadidi, AmirSilva, CarlaJain, EraSilva, CláudiaJanarthanan, GopinathanSimionescu, AgnetaJayakumar, RangasamySingh, Devinder P.Jayasuriya, Chathuraka T.Sinha, Michael S.Jin, BoSivathanu, VivekJohansson, BjörnSkruzny, MichalJones, Elena A.Sokolis, Dimitrios P.Joshi-Navare, KasturiSokołowska, EmiliaJung, Kee WookSon, KeunBaDaKalfa, DavidSong, ChunhuaKamavuako, ErnestSong, Ping’anKan, EdwinSousa, AurelianaKasoju, NareshSousa, Susana R.Katsimpouras, ConstantinosSreerama, LakshmaiahKengelbach-Weigand, AnnikaStobiecka, MagdalenaKeshavarz, MeysamStock, PeggyKessi-Perez, EduardoStoyanov, JivkoKhedoe, Padmini S.J.Tabuchi, MasashiKhodabukus, AlastairTaina, TervahautaKhosroshahi, Siamak FarajzadehTakayama, KazuoKim, Dong-HyunTan, Hsih-YinKim, Ik-HwanTanjore, DeeptiKim, JinsikTaouk, BecharaKim, SejoongTariq, MadihaKittel-Schneider, SarahTavelli, LorenzoKlein, StefanieTeo, JeremyKlekiel, TomaszTerry, BenjaminKlimek, KatarzynaTerzini, MaraKluger, Petra J.Thomas, RobertKohla, GuidoTiriac, HerveKohn, Julie C.Tobet, StuartKoledova, ZuzanaTorres-Perez, Jose VicenteKominek, JacekTosi, DanieleKoutras, AthanasiosToxiri, StefanoKovács, PéterTrevathan, JamesKregiel, Dorota MariaTripisciano, CarlaKremen, VaclavTripodi, MarcoKruithof, BoudewijnTruong, Danh D.Kuang, XiaoTsafrakidou, PanagiotaKulda, VlastimilTsouti, VasilikiKumar, BrajeshUngi, TamasKung, EthanUosaki, HidekiKurada, LalithaUrtnasan, ErdenebayarKurniawan, Nicholas AgungUsack, JosephKurreck, JensVan Beuningen, HenkLand, RüdigerVan Grunsven, LeoLangenderfer, JosephVan Wyk, NielLanger, Jerzy J.Vannier, MichaelLee, Chang HunVassileva, ElenaLee, HyeongjinVaughan, MelLee, Jin-HoVelázquez Blázquez, José SebastiánLee, Lik ChuanVerhulst, StefaanLee, Myung-ShinVidic, JasminaLehocký, MariánVilela, AliceLemaigre, FrederiqueVillalba-Mora, ElenaLewis, PhillipVining, Kyle HolmbergL’Heureux, NicolasWan, XiaoLi, ZhongWang, XiaojuLi, FengWard, Alastair JamesLi, Jiao JiaoWhitehead, Timothy A.Li, Jing-ShengWilson, Heather L.Li, PingWolf, Matthew TLi, Shengwen CalvinWu, HuiquanLi, XinWu, YangLiao, PanWystalska, KatarzynaLim, DanielXia, ChangleiLim, Jung YulXiao, LiLin, Chih-HsunXu, ChuanLin, LongXu, FeiLincoln, RichardXu, PengLiu, Cheng-HsienXue, XufengLiu, ZhenqiuYadav, Pramod KumarLluna, Amparo GameroYadavalli, Nataraja SekharLongo, AngelaYallapu, Murali MohanLopez, RandolphYang, Kai-ChiangLorenzo, MicheleYang, Hyeong-CheolLou, Yan-RuYavits, LeonidLow, LucieYavuz, MetinLu, FengYazdi, ImanMa, XiuquanYe, HaihangMachluf, MarcelleYe, LeiMadduri, SrinivasYou, JingjingMaestro, BeatrizYu, S. MichaelMagdassi, ShlomoYuan, XuegangMagyari, KlaraZambelli, TomasoMajchrzak, EwaZambrano, Byron A.Mak, MichaelZarnowska, Iwona MariaMakarevich, PavelZhang, BingMallis, PanagiotisZhang, DianbaoMandatori, DomitillaZhang, YiMarek, KieliszekZhu, JunjunMartin, Andrew Read


